# A high-glucose diet affects Achilles tendon healing in rats

**DOI:** 10.1038/s41598-017-00700-z

**Published:** 2017-04-10

**Authors:** Stefanie Korntner, Nadja Kunkel, Christine Lehner, Renate Gehwolf, Andrea Wagner, Peter Augat, Daniel Stephan, Verena Heu, Hans-Christian Bauer, Andreas Traweger, Herbert Tempfer

**Affiliations:** 1 Institute of Tendon & Bone Regeneration, Paracelsus Medical University Salzburg, Spinal Cord Injury and Tissue Regeneration Centre Salzburg, Salzburg, AT Austria; 2 Austrian Cluster for Tissue Regeneration, Vienna, AT Austria; 3 University Hospital of Salzburg, Department of Trauma Surgery and Sports Injuries, Salzburg, AT Austria; 4 Institute of Biomechanics, Trauma Center Murnau, Murnau, DE Germany; 5 University Hospital of Salzburg, Department of Paediatrics, Salzburg, AT Austria

## Abstract

Chronic and acute tendinopathies are difficult to treat and tendon healing is generally a very slow and incomplete process and our general understanding of tendon biology and regeneration lags behind that of muscle or bone. Although still largely unexplored, several studies suggest a positive effect of nutritional interventions on tendon health and repair. With this study, we aim to reveal effects of a high-glucose diet on tendon neoformation in a non-diabetic rat model of Achilles tenotomy. After surgery animals received either a high-glucose diet or a control diet for 2 and 4 weeks, respectively. Compared to the control group, tendon repair tissue thickness and stiffness were increased in the high-glucose group after 2 weeks and gait pattern was altered after 1 and 2 weeks. Cell proliferation was up to 3-fold higher and the expression of the chondrogenic marker genes *Sox9*, *Col2a1*, *Acan* and *Comp* was significantly increased 2 and 4 weeks post-surgery. Further, a moderate increase in cartilage-like areas within the repair tissue was evident after 4 weeks of a high-glucose diet regimen. In summary, we propose that a high-glucose diet significantly affects tendon healing after injury in non-diabetic rats, potentially driving chondrogenic degeneration.

## Introduction

Tendon injuries are caused by a variety of intrinsic and extrinsic factors such as insufficient loading, local inflammation and individual factors like age^1, 2^. Due to the low tissue turnover and cell proliferation rate, low vessel and nerve supply^3–5^, tendon repair is generally a slow process resulting in the formation of a mechanically inferior scar tissue often leading to a decreased range of motion, pain, and an increased risk of a recurrent rupture^6–9^. Although the impact of various intrinsic and extrinsic factors affecting tendon health and quality are subject of intense research, relatively few studies have investigated the relationship between nutrition and tendon healing. Ömeroğlu, *et al*.^10^ reported that a high dose oral vitamin C supplementation accelerated rat Achilles tendon healing and another study suggests a positive impact on tendon-to-bone healing in rabbits after oral glucosamine/chondroitin sulphate supplementation^11^. However, little attention has been paid to the potential effects of short-term blood glucose elevations by nutritional glucose in early tendon healing in non-diabetic individuals. Interestingly, Eliasson, *et al*.^12^ reported an elevated metabolic activity, particularly glucose uptake, in ruptured Achilles tendons up to 1 year after surgical repair. Further, we previously described a tendon cell population in human and rat tendons, which upon glucose stimulation secretes insulin^13^. Taken together, these findings indicate a potential role of glucose in tendon pathologies and tendon healing, also in non-diabetic individuals. Clinically, hyperosmolar dextrose solutions together with an anaesthetic are often injected into or alongside tendons and ligaments aiming at reducing chronic pain and to stimulate cell proliferation and extracellular matrix (ECM) deposition by inducing an inflammatory response^14^. Generally, this increasingly popular treatment for a wide range of musculoskeletal pathologies is deemed safe, however evidence to support the effectiveness of this so-called prolotherapy is limited as only few randomised controlled clinical trials have been conducted^15^. Further, little is known about the long-term molecular and cellular ramifications on tendon quality following prolotherapy.

The objective of this study was to evaluate the effect of glucose-rich nutrition on Achilles tendon healing through gait, histological, biomechanical, and molecular analyses in a rat model. Our findings reveal that a high-glucose diet indeed has a favourable effect on cell proliferation and ECM deposition within the newly formed tissue, however potentially increases the risk of chondroid degeneration in injured tendons.

## Methods

### Animal study design

All animal experiments and procedures were conducted in accordance with Austrian laws on animal experimentation and were approved by Austrian regulatory authorities on animal experimentation (permit numbers 20901-TVG/62/4-2013 and BMWFW-66.012/0035-WF/V/3b/2014). A total of 60 female Lewis rats aged 2 months were included in the study. A 2 mm full-thickness transverse Achilles tenotomy was created on the right hind limb of each animal. The tendon was transversally excised and 2 mm in proximal/distal extension were removed 3 mm proximal of the calcaneus, resulting in a complete separation of the proximal and distal segments. Tendons were left unsutured and only the skin was sutured (SI Fig. 1, see also SI methods). Immediately after surgery the feeding regimen commenced and the experimental groups received either a control diet or a high-glucose diet (ssniff® EF R/M High glucose), containing an elevated total content of carbohydrates composed of glucose instead of long chain carbohydrates (SI Table 1), for a period of 2 (control group: n = 24/high-glucose group: n = 25) and 4 weeks (control group: n = 5/high-glucose group: n = 6), respectively (see also SI Tables 2 and 3). The control diet was composed as follows: 61 kJ % carbohydrates (46.8 starch, 10.8 sugar), 30 kJ % protein and 9 kJ % fat. The high-glucose diet was composed as follows: 67 kJ % carbohydrates (50.0 glucose, 12.0 dextrines), 27 kJ % protein and 6 kJ % fat.

The animals were weighed prior to and 1, 2, 3 and 4 weeks after surgery (SI Fig. 2a,b) and blood glucose was measured prior to and 2 and 4 weeks after surgery (SI Fig. 2b). All animals were housed in groups and were provided access to food and water *ad libitum*. After a period of 2 weeks animals were euthanised and after tendon size measurements, tendon repair tissue as well as contralateral control tendons were harvested and either stored at −20 °C for biomechanical testing, immersion-fixed in phosphate-buffered saline (PBS) containing 4% paraformaldehyde for histologic analysis or stored in RNAlater (RNA stabilising reagent, Quiagen, Germany) for gene expression analysis. Additionally, after a period of 4 weeks, animals were euthanised and blood samples of animals of the control group (n = 5) and of the high-glucose group (n = 5) were collected by cardiac puncture into tubes containing EDTA. Blood samples were used to determine glycated haemoglobin A1c (HbA1c) using a rat HbA1c-kit (Cat. No. 80300, Crystal Chem INC, Spain) according to the manufacturer’s instructions (SI Fig. 2d). After tendon size measurements *in situ*, tendon repair tissue as well as contralateral control tendons were collected for histologic analysis and gene expression analysis.

### Gait analysis

The gait of animals receiving a control diet for 2 weeks (n = 19) or a high-glucose diet for 2 weeks (n = 20) was analysed using the CatWalk system (CatWalk XT; Noldus Information Technology, Wageningen, The Netherlands). Gait analysis was conducted once pre-surgery and 1 and 2 weeks after Achilles tenotomy (see also SI methods). To analyse the degree of weight-bearing of the injured limb, the Intermediate Toe Spread (ITS) was measured, representing the distance between the second and fourth toe of the hind limb. ITS is considered as a sensitive and reliable indicator of Achilles tendon function and is a measure of the ability of the rat to load and hence spread its middle three toes^16, 17^.

### Tissue harvesting and gross morphology

2 weeks and 4 weeks after Achilles tenotomy, animals were euthanised by CO_2_ inhalation and the Achilles tendons, both left and right, were dissected. Before tendon harvesting, tendon thickness and length were measured using a digital calliper. Thickness was determined 3 mm proximal from the calcaneus. As tendon length, the distance from the calcaneal insertion to the distal most insertion of the muscle was considered (SI Fig. 4). For comparison of tissue measurements with intact controls, contralateral tendons of the control groups were used.

### Biomechanical testing

After 2 weeks, Achilles tendon repair tissues as well as contralateral control tendons of the control group (n = 14) and the high-glucose group (n = 14) were harvested and tested on a universal material testing machine (Zwick, Ulm, Germany) at 15 ° loading angle at 0.1 mm/min until failure immediately after a preload of 0.5 N had been applied (see also SI methods). Maximum tensile load was defined as the maximum force at tendon failure and is expressed in newton (N). Tendon stiffness was calculated from the linear proportion of the force/elongation curve and is expressed as N/mm.

### Histology and immunohistochemistry

For histologic analyses, Achilles tendon repair tissues and contralateral tendons of all experimental groups (n = 3/group) were dissected from the proximal musculo-tendinous junction to the distal tendon-to-bone attachment zone and processed as described in Tempfer, *et al*.^4^. For descriptive histology 7 µm thick cryosections were stained either using Hematoxylin & Eosin stain (data not shown), Masson-Goldner’s trichrome stain or Herovici’s polychrome stain according to the manufacturer’s guidelines (see SI methods for further details). Areas of chondrification within the defect region 4 weeks after surgery were determined measuring Safranin O stained areas using ImageJ (v. 1.46). For each repair tissue 3 different frontal-longitudinal sections were analysed (1 section of the middle part, 1 section ventral of the middle part and 1 section dorsal of the middle part). For each section 5 consecutive images were captured spanning the entire length of the repair tissue, omitting the transition zones of original tendon stumps to repair tissue.

Immunohistochemical detection of cells positive for KI67 and staining for Collagen type 1 (COL1) and Collagen type 3 (COL3) was performed on 30 µm thick cryosections of the repair tissues. Slides were incubated overnight at 4 °C, with the primary antibody (monoclonal rabbit anti-KI67, RM-9106-S0, Thermo Fisher Scientific; polyclonal rabbit anti-Collagen1, ab34710, Abcam; polyclonal rabbit anti-Collagen 3, ab7778, Abcam) in PBS containing 10% donkey serum (Sigma-Aldrich, Wien, Austria), 1% bovine serum albumin (BSA; Sigma-Aldrich), and 0.5% Triton X-100 (Merck, Darmstadt, Germany) and binding sites of primary antibodies were visualised by a corresponding Alexa568-tagged antiserum (donkey anti rabbit Alexa Fluor 568, Life Technologies, Vienna, Austria). Nuclear staining was performed using 4′,6-Diamidino-2 phenylindol dihydroclorid (DAPI). For every section a total of 5 non-overlapping regions within the repair tissue area were randomly chosen. Total numbers of DAPI-positive as well as KI67-positive cells were counted using ImageJ and the percentages of immune-positive cells were calculated as well as cell number/mm^2^ was determined.

### Quantitative RT-PCR

Total RNA was isolated from tendon repair tissues of the control diet group and the high-glucose diet group 2 (control group: n = 5/high-glucose group: n = 5) and 4 weeks (control group: n = 3/high-glucose group: n = 3) after surgery and of intact control tendons (n = 3) using TRI® Reagent (Sigma-Aldrich; Vienna, Austria) according to the manufacturer’s protocol (see also SI methods for details). qRT-PCR was performed as described in Lehner, *et al*.^18^, using TaqMan® assays (Integrated DNA Technologies, Coralville, IA, USA) targeting *Sox9* (SRY box 9), *Col2a1* (collagen, type II, alpha 1), *Acan* (aggrecan), *Comp* (cartilage oligomeric matrix protein), *Fabp2* (fatty acid binding protein 2), *Pparγ* (peroxisome proliferator-activated receptor γ), *Runx2* (runt-related transcription factor 2), *Col1a1* (collagen type 1 α1), *Col3a1* (collagen type 3 α1), *Scx* (scleraxis), *Mkx* (mohawk), *Tnmd* (tenomodulin), *Il1b* (interleukin 1b), *Il6* (interleukin 6), *Il10* (interleukin 10) (see also SI methods for details).

### Statistical analysis

Statistical analyses were performed using GraphPad Prism v.5.04 (La Jolla, CA, USA). Numerical data is presented as means ± standard deviation. One way analysis of variance (ANOVA) for multiple comparisons and 2-sample t-test for pair-wise comparisons were employed after confirming normal distribution of the data (D’Agostino and Pearson omnibus normality test). Non-parametric statistics were utilised when the above assumption was violated and consequently Kruskal–Wallis test for multiple comparisons or Mann–Whitney test to determine two-tailed p-value samples was carried out. Statistical significance was set at α = 0.05.

## Results

### General animal health and metabolic parameters

All animals showed normal behaviour, with non-restricted weight bearing 48 h after surgery. However, one animal of both control groups died during surgery due to unknown reasons. For none of the animals the body weight was significantly increased after receiving either the control or the high-glucose diet for 2 or 4 weeks (SI Fig. 2a,b). Therefore, it can be assumed that the caloric intake was comparable for both treatment groups. Blood glucose levels of animals receiving a high-glucose diet were in the normal range of non-diabetic individuals after 2 and 4 weeks of feeding regimen and comparable with the control diet group (SI Fig. 2c). HbA1c levels, reflecting the glycosylation of haemoglobin in erythrocytes and hence long-term glucose control did also not differ between the dietary groups after a feeding regimen of 4 weeks (SI Fig. 2d). HbA1c was 6.3 ± 1.0% for the control group and 6.2 ± 0.4% for the high-glucose group (p = 0.90). As expected, normal body weight together with physiological levels of blood glucose and no elevation in % HbA1c demonstrates that 4 weeks of a high-glucose diet did not result in any signs of diabetes.

### Gait analysis

**Figure 1:**
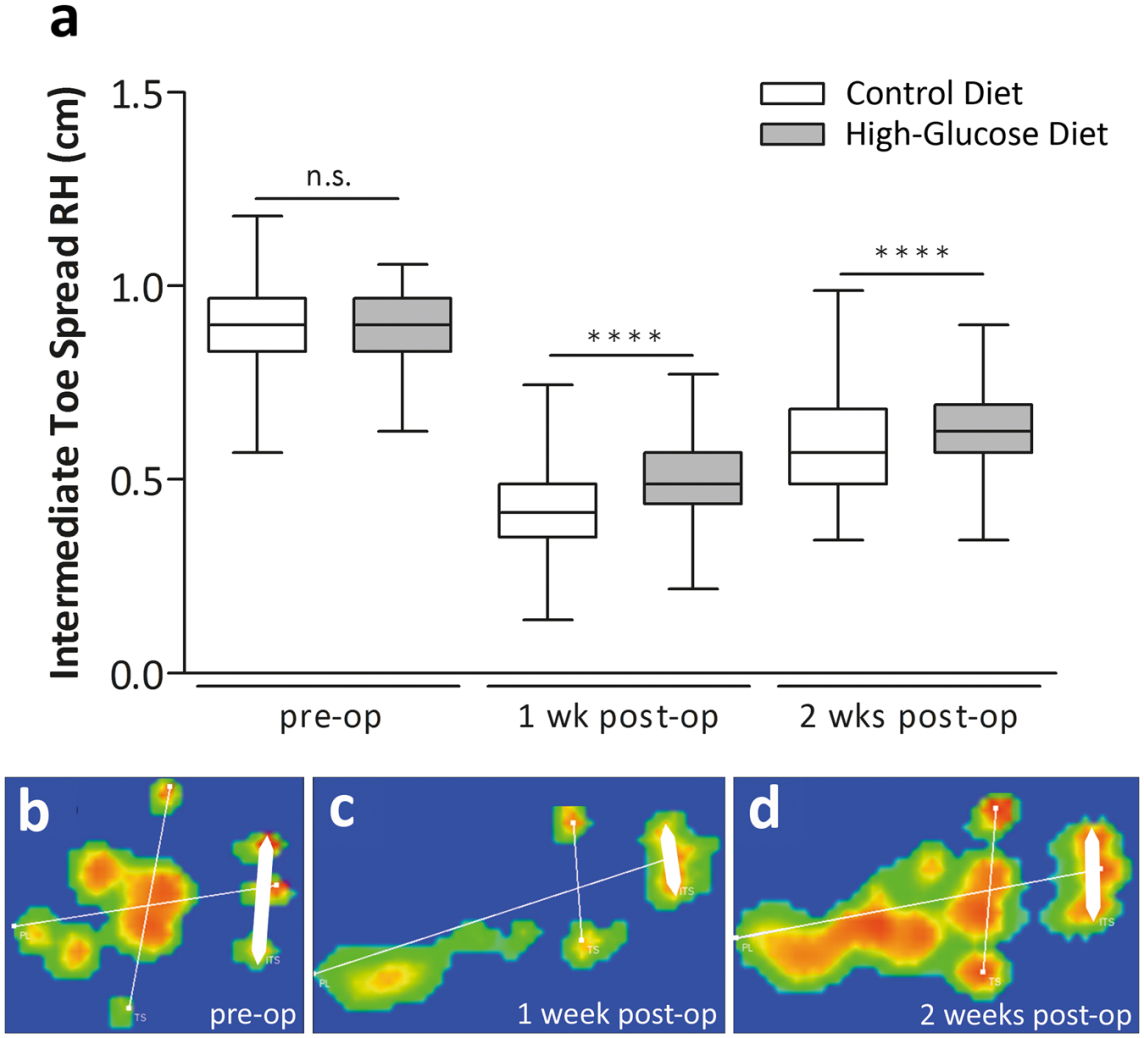
Gait Analysis: Intermediate toe spread (ITS). Intermediate toe spread (ITS) of the right hind limb (RH) before surgery was equal between the experimental groups (**a**). One week post-op, ITS was significantly higher in the high-glucose group compared to the control group (p < 0.0001; Mann-Whitney test, two-tailed). 2 weeks post-op, ITS remained significantly higher in the high-glucose group compared to the control diet group (p < 0.0001; Mann-Whitney test, two-tailed). Representative images for ITS measurements pre-op (**b**, one week post-op (**c**) and 2 weeks post-op (**d**). ITS (thick white bar) represents the distance between second and fourth toe.

ITS of the right hind limb (RH) before surgery (all animals were fed control diet) were similar for both groups (Fig. 1a; p > 0.05), being 0.90 ± 0.10 cm for the control animals (n = 251 footprints, 19 animals) and 0.906 ± 0.09 cm for the high-glucose group (n = 237 footprints, 20 animals). One week after surgery, for animals receiving the high-glucose diet the ITS of the RH was significantly higher (p < 0.0001; 0.50 ± 0.10 cm; n = 448 footprints, 20 animals) when compared to the control animals (0.42 ± 0.11 cm; n = 528 footprints, 19 animals). Similarly, two weeks after surgery, ITS was significantly higher (p < 0.0001) in the high-glucose diet group (0.64 ± 0.09 cm; n = 493 footprints, 20 animals), compared to animals receiving a control diet for 2 weeks (0.60 ± 0.11 cm; n = 505 footprints, 19 animals). Taken together, for animals with an increased glucose uptake for 2 weeks an increased ITS was recorded one and two weeks after surgery, indicating a higher load of the hind limb and most likely less pain and/or better mechanical properties, respectively.

### Tendon size measurements

**Figure 2:**
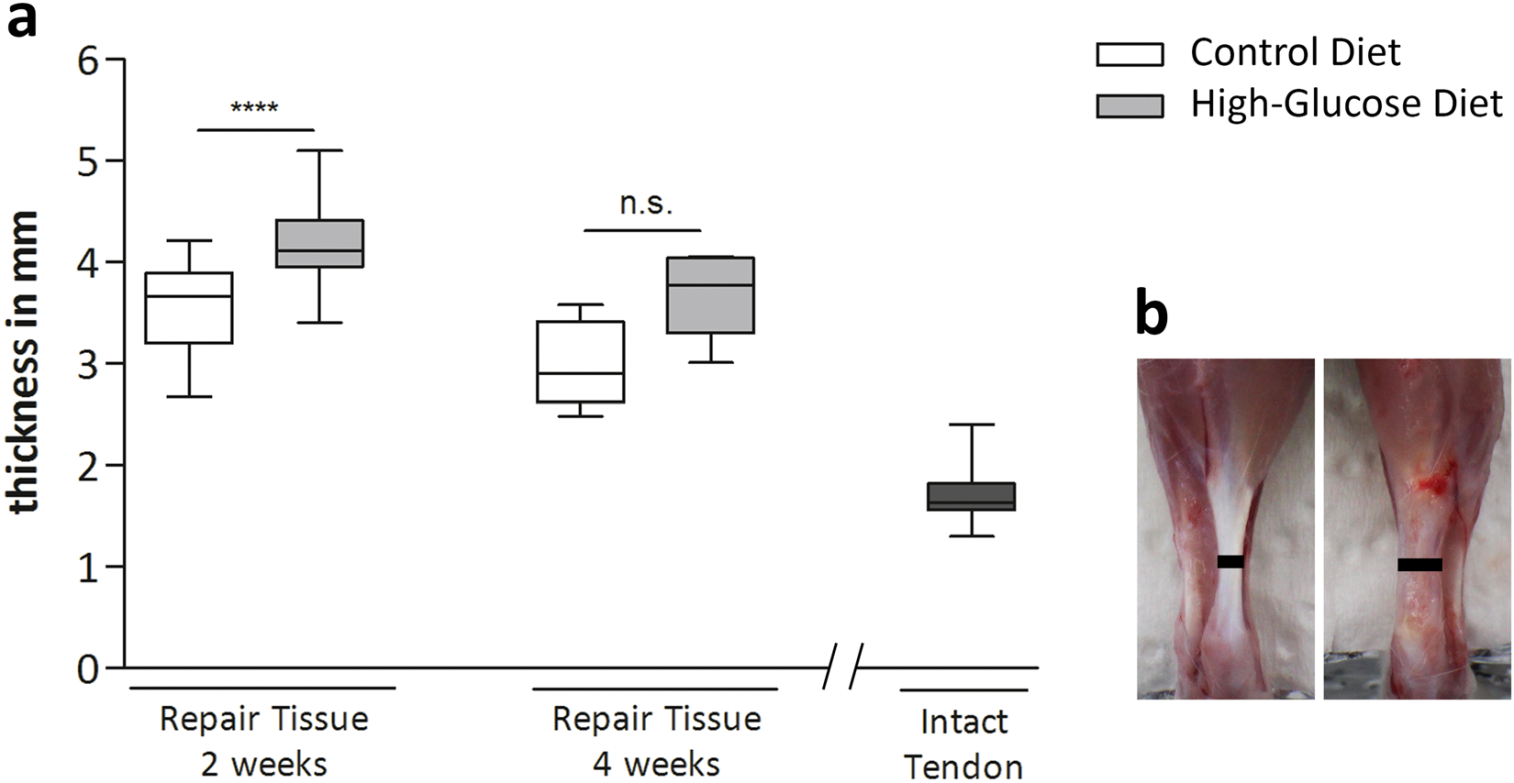
Repair tissue thickness measurements. Comparisons among the experimental groups 2 weeks after surgery revealed significantly increased thickness in the high-glucose group (n = 25), compared to the control diet group (n = 24, p < 0.0001; Mann-Whitney test, two-tailed) (**a**). 4 weeks after surgery no significant difference could be detected (n = 6; p = 0.0519; Mann-Whitney test, two-tailed). Tendon thickness was determined 3 mm proximal from the calcaneus (**b**, black bars). Values for intact tendons are provided as a reference (n = 24).

After 2 weeks, the mid-portion of the tendon repair tissue was significantly thicker (p < 0.001) for each experimental group when compared to the intact tendons (Fig. 2a,b). Comparisons among the diet groups revealed significantly increased thickness in animals receiving a high-glucose diet for 2 weeks compared to the control group (p < 0.0001). Thickness was 3.55 ± 0.45 mm (n = 24) for the control diet group, 4.15 ± 0.36 mm for the high-glucose diet group (n = 25), and 1.73 ± 0.21 mm (n = 24) for intact control tendons. After 4 weeks, thickness of the repair tissues of the high-glucose group remained significantly increased compared to the intact contralateral tendons of the control group (p < 0.01) and were moderately, but statistically not significantly thicker compared to the control group (p = 0.0519). Repair tissue thickness was 2.99 ± 0.43 mm (n = 5) for the control group and 3.67 ± 0.42 mm (n = 6) for the high-glucose group (Fig. 2b). In summary, a high-glucose diet leads to increased repair tissue thickness 2 weeks after surgery compared to a control diet.

### Biomechanical Testing

**Figure 3:**
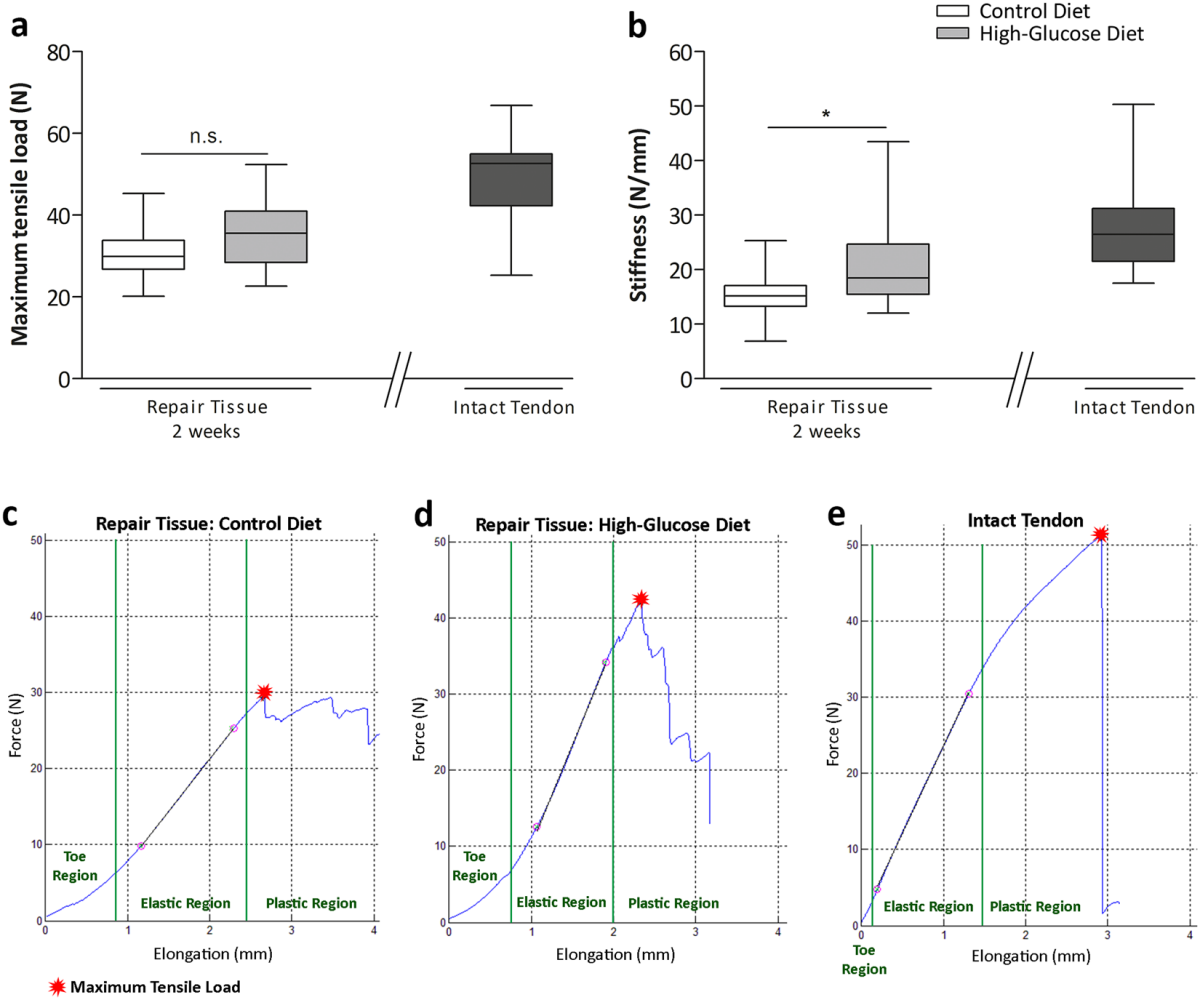
Biomechanical properties of tendon repair tissues 2 weeks after Achilles tenotomy. Biomechanical tests comparing maximum tensile load of the repair tissues 2 weeks after surgery revealed no significant difference (p > 0.05; Mann-Whitney test, two-tailed) between the experimental groups (**a**). Tendon stiffness measurements 2 weeks after surgery among the experimental groups showed significantly stiffer repair tissues (p < 0.05, asterisk; Mann-Whitney test, two-tailed) in the high-glucose group (n = 14) when compared to the control group (n = 14) (**b**). Values for intact tendons are provided as a reference (n = 9). Representative force-elongation diagrams of the repair tissues of the control diet group (**c**), the high-glucose diet group (**d**) and of an intact tendon (**e**). In the toe-region collagen fibres are crimped and become elongated and straightened with applied loading. The linear region represents tendon stiffness. Microscopic tears occur in the plastic region where the slope drops until the maximum tensile load is reached and the tendon ruptures.

Comparisons of maximum tensile load of the repair tissues 2 weeks after surgery revealed no statistically significant difference between the experimental groups. Maximum tensile load was 30.9 ± 6.4 N (n = 14) for repair tissues of animals receiving a control diet and 35.4 ± 8.8 N (n = 14) for the high-glucose diet group (Fig. 3a). However, repair tissue stiffness of the high-glucose group was significantly higher compared to tissues harvested from animals that had received the control diet (p < 0.05). Two weeks post-surgery tissue stiffness for the control group was 15.1 ± 4.3 N/mm^2^ (n = 14) and 20.8 ± 8.1 N/mm^2^ (n = 14) for the high-glucose diet group (Fig. 3b). Taken together, a high-glucose diet did not result in an altered tensile strength of the repair tissue, however the stiffness was significantly increased.

### Descriptive histology and tenogenic gene expression

**Figure 4:**
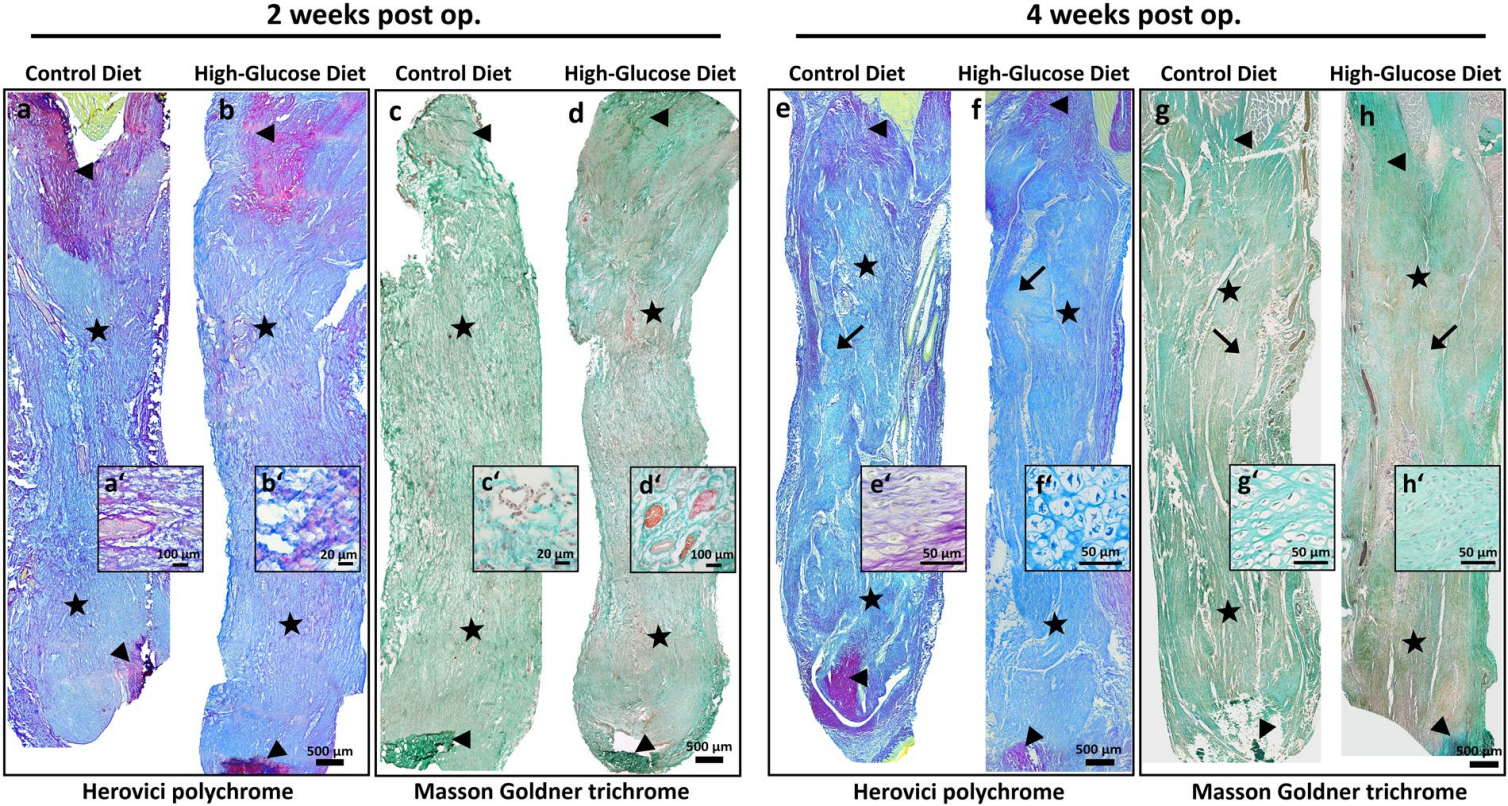
Descriptive histology of tendon repair tissues 2 and 4 weeks post-surgery. Representative histologic images of tendon repair tissue sections of the control diet group and the high-glucose diet group stained with either Herovici’s polychrome (**a**,**b**,**e**,**f**), or Masson-Goldner trichrome (**c**,**d**,**g**,**h**). Loose collagen fibre orientation (c’), hypercellularity and ingrowth of vessels and nerves after 2 weeks (d’). The extracellular matrix (ECM) was mainly composed of immature collagen, most likely corresponding to collagen type III ((**a**,**b**,**e**,**f**); blue staining), both 2 and 4 weeks after surgery. Aggregates of cells with a chondrogenic phenotype (*arrows*) were frequently observed 4 weeks post-surgery in both the control and the high-glucose diet group, although to a different extent (g’,h’). No difference could be detected between the experimental groups. *Arrows*: retracted proximal and distal ends of the original tendon; *asterisks*: newly formed tendon repair tissue.

Representative histological sections of the repair tissues (Fig. 4) stained with either Herovici’s polychrome (Fig. 4a,b,e,f), or Masson-Goldner trichrome (Fig. 4c,d,g,h) showed a complete bridging of the tendon defect area (*asterisks*) 2 and 4 weeks after surgery. The fibrous repair tissue showed loose fibre orientation and an overall heterogeneous texture after 2 and 4 weeks (Fig. 4b’,c’), with increased cellularity and ingrowth of vessels and nerves (Fig. 4a’,d’) and no gross differences between the experimental groups was evident. The extracellular matrix (ECM) of the newly formed repair tissue was mainly composed of immature collagen, most likely corresponding to collagen type III (Fig. 4a,b,e,f; blue staining), both 2 and 4 weeks after surgery. 4 weeks after surgery the repair tissue still appeared rather heterogeneous and aggregates of cells with a chondrogenic phenotype (*arrows*) were frequently observed for the control and treatment group, although to a different extent (Fig. 4e’,f’,g’,h’).

**Figure 5:**
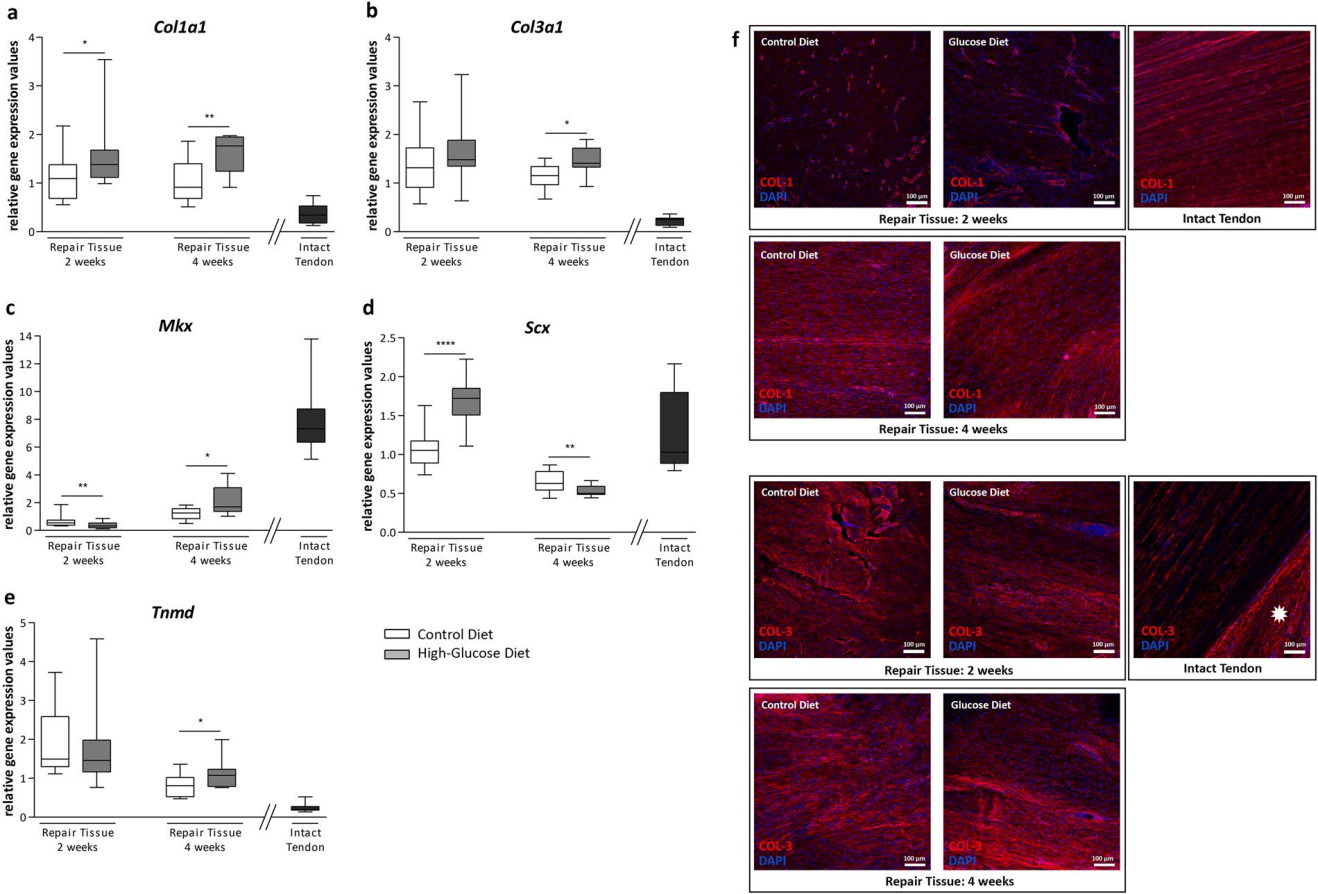
Analysis of COL1 and COL3 and tenogenic marker gene expression. Quantitative PCR analysis of *Col1a1* (**a**) and *Col3a1* (**b**) and the tenogenic lineage associated genes *Mkx* (**c**), *Scx* (**d**), and *Tnmd* (**e**) in the repair tissues of the control diet group and the high-glucose diet group after 2 and 4 weeks. COL1 staining was mostly confined to capillaries within the repair tissue after 2 weeks whereas a deposition of collagen type 1 in the extracellular matrix of the repair tissue was observed after 4 weeks. Strong COL1 staining in intact tendon tissue is shown as a reference (**f**). Substantial collagen type 3 deposition in the extracellular matrix was seen after 2 and 4 weeks. Note the strong COL3 staining in the epitenon (*asterisk*) of intact tendon tissue (**g**). Compared to intact tendons, *Tnmd* expression was increased 2 and 4 weeks after surgery. *Mkx* expression was lower in the repair tissues, showing no substantial change over time. *Scx* expression 2 weeks post-tenotomy was comparable to intact tendons. 4 weeks post-surgery *Scx* transcript levels were lower, the decrease being strongest for the high-glucose group. Values for intact tendons are provided as a reference (n = 12).

Gene expression of Col1a1 and Col3a1 was moderately increased in the high-glucose diet group after 2 and 4 weeks (less than 2-fold). Immunohistochemistry revealed cytoplasmic COL1 (collagen type 1) mainly in the vicinity of capillaries, whereas substantial deposition of COL3 (collagen type 3) in the extracellular matrix (ECM) was evident in the repair tissues of both groups. In contrast, 4 weeks post-surgery robust COL1 and COL3 ECM-deposition could be detected in the ECM of the repair tissues of both diet groups (Fig. 5).

Differences in gene expression levels of the tendon-associated genes mohawk (*Mkx*), scleraxis (*Scx*) and tenomodulin (*Tnmd*) were generally less than 2-fold within the two diet groups. Compared to the intact contralateral tendons, *Tnmd* expression was increased 2 and 4 weeks after surgery. In contrast, mRNA levels of *Mkx* were lower in the repair tissues, showing no substantial change over time. Finally, *Scx* expression 2 weeks post-tenotomy was comparable to the contralateral tendons. 4 weeks post-surgery *Scx* transcript levels were lower, the decrease being strongest for the high-glucose group.

### Cell proliferation and cell number

**Figure 6:**
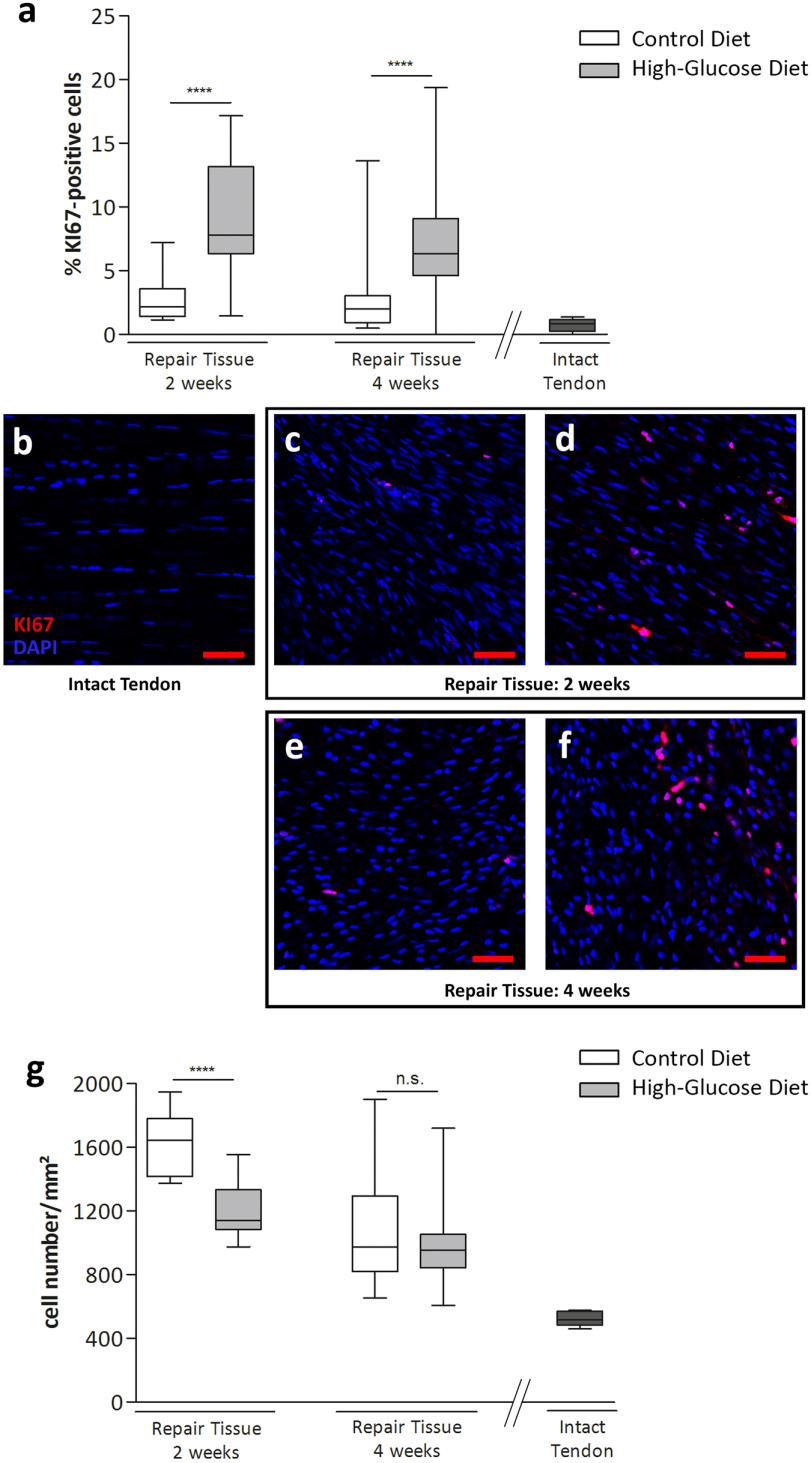
Quantification of cell proliferation by KI67 in tendon repair tissues. Compared to a control diet group, a high-glucose diet significantly increases the proportion of KI67-positive cells after 2 (p < 0.0001, n = 15 images of 3 animals/group) and 4 weeks (p < 0.0001, n = 30 images of 3 animals/group) (**a**) (Mann-Whitney test, two-tailed), whereas the total number of cells/area is significantly decreased in the high-glucose group compared to the control group after 2 weeks (**g**) (p < 0.0001, Mann-Whitney test, two-tailed). Representative images for intact control tendons (**b**) and tendon repair tissues 2 weeks post op: control group (**c**), high-glucose group (**d**) and 4 weeks post op: control group (**e**) and high-glucose group (**f**). Values for intact tendons are provided as a reference (n = 5 images of 3 animals).

Quantification of KI67-positive cells revealed a significant increase within the repair tissues of animals receiving a high-glucose diet for 2 (p < 0.0001) and 4 weeks (p < 0.0001) (Fig. 6a), whereas the total number of cells/area was significantly decreased (p < 0.0001) in the high-glucose diet group after 2 weeks. However, 4 weeks post-surgery the latter was not the case anymore (p > 0.05; Fig. 6g). 2 weeks after surgery, the percentage of KI67-positive cells was 3.0 ± 1.9% (n = 15 images from 3 animals) for the control group and 9.1 ± 4.5% (n = 15) for the high-glucose group. 4 weeks after surgery 2.7 ± 2.8% (n = 30) were determined for the control group and 7.4 ± 4.5% (n = 45) for the high glucose group. After 2 weeks cell number/mm^2^ was 1631 ± 187 (n = 15) and 1189 ± 163 (n = 15) for the control and the glucose diet group, respectively. 4 weeks post-tenotomy 1060 ± 327 (control; n = 30) and 968 ± 218 (high-glucose; n = 45) were determined.

### Gene expression analysis and histomorphometry

**Figure 7:**
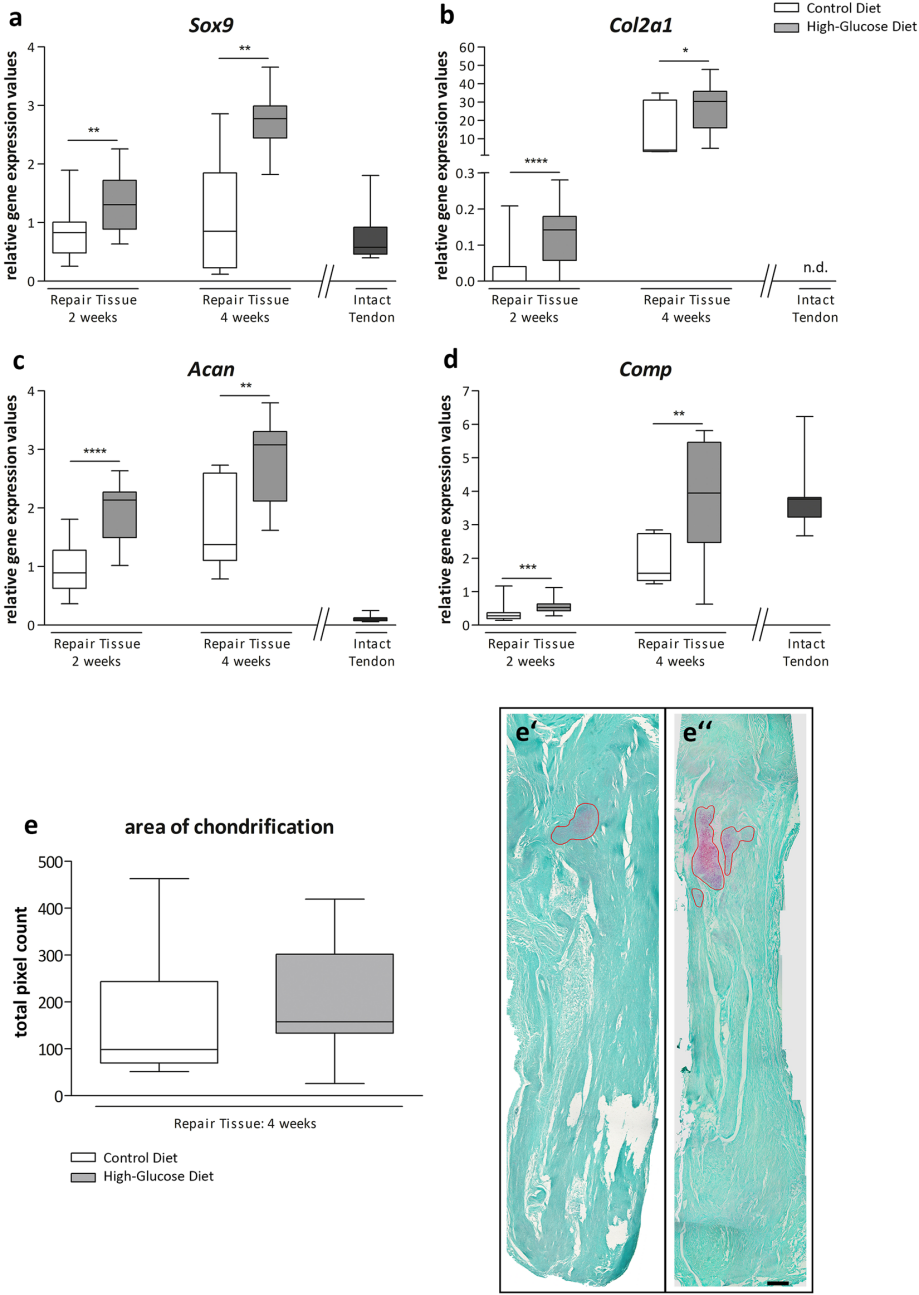
Gene expression analysis and histomorphometry of tendon repair tissues. Quantitative PCR analysis of tendon repair tissues shows that the chondrocyte lineage-associated genes *Sox9* (**a**), *Col2a1* (**b**), *Acan* (**c**) and *Comp* (**d**) are significantly upregulated in animals receiving a high-glucose diet for 2 and 4 weeks, respectively, compared to animals fed a control diet (Mann-Whitney test, two-tailed). Values for intact tendons are provided as a reference. Quantification of areas showing beginning chondrification 4 weeks after surgery did show a trend of increasing chondrification in the high-glucose group (**e**), though the difference was not significant (p = 0,328; Mann-Whitney test, two-tailed). Representative Safranin O & Fast green stained sections of the control diet group (e’) and the high-glucose diet group 4 weeks post op. (e”). Scale bar: 500 µm.

Comparisons of the experimental groups showed a significant increase of *Sox9* expression in tendon-repair tissue harvested from animals receiving a high-glucose diet, both 2 (p < 0.01) and 4 (p < 0.01) weeks after surgery (Fig. 7a). 2 weeks after surgery *Col2a1* mRNA levels were also significantly increased (p < 0.0001) with a further strong increase after 4 weeks, showing a significant difference between the diet groups (p < 0.05) (Fig. 7b). As expected, no *Col2a1* expression was detectable in intact control tendons. Similarly, *Acan* expression was significantly increased in the high-glucose group after 2 weeks (p < 0.0001) and was further upregulated after 4 weeks (p < 0.01) when compared to the control group (Fig. 7c). 2 weeks (p < 0.001) and 4 weeks (p < 0.01) post-surgery, gene expression of *Comp* was significantly increased in the high-glucose group when compared to the control group (Fig. 7d). Compared to the intact contralateral tendon, *Comp* levels were restored 4 weeks post-surgery. Taken together, these data strongly suggest an increase in genes driving chondrogenesis. In contrast, there was no meaningful difference observed for adipogenesis- and osteogenesis-associated marker genes (*Fabp2*, *Pparg*, *Runx2*) within the treatment groups or in comparison to the intact contralateral tendons 2 and 4 weeks after surgery (SI Fig. 4).

Histomorphometric quantification of Safranin O stained areas revealed deposition of glycosaminoglycans typical for chondrification 4 weeks after surgery (Fig. 7e’,e”). For the high-glucose diet group a moderate increase in Safranin O–positive areas was evident, however the difference was statistically not significant (p = 0.3277) (Fig. 7e). Areas showing beginning chondrifications were 159.6 ± 154.4 (pixels per area) for the control group (n = 6 sections of 2 animals) and 199.4 ± 126.5 for the high-glucose group (n = 9 sections of 3 animals).

Taken together, both on the mRNA level and the phenotypic level an increase in early stage chondrogenesis was evident for Achilles tendon repair tissue harvested form animals that had received a high-glucose diet for 2 and 4 weeks, respectively.

## Discussion

The ideal therapy for chronic and acute tendon injuries remains unclear and new treatment modalities are needed to improve tendon healing or even enable full functional restoration. More recently, the impact of nutritional supplements to further improve tendon healing in combination with other interventions is being investigated^19^. Generally, it is well known that appropriate nutrition is important for physiological wound healing to occur in various tissues and adequate amounts of glucose are mandatory for providing energy for angiogenesis and deposition of new tissue^20, 21^. With this study we show for the first time that nutritional glucose significantly affects tendon healing in a non-diabetic rat model. While overall no gross changes in the gait pattern were observed, the intermediate toe spread (ITS), a surrogate for limb loading^16, 17^, was significantly increased for animals that had received a high-glucose diet potentially indicating improved mechanical properties and/or less pain^22^. Along these lines we also observed an increase in tissue stiffness and thickness after high-glucose treatment. Even though this study is not explicitly designed to examine the mechanisms underlying the so called prolotherapy or proliferation therapy, our data are well in line with the effects described for this therapy. Thereby mild irritants, such as a hyperosmolar dextrose solution is injected in combination with an anaesthetic to treat chronic and acute ailments of tendons and ligaments. Besides the assumption that glucose might provide a locally concentrated energy supply, prolotherapy is hypothesised to induce a host inflammatory response, which leads to formation and deposition of new collagen and finally results in biomechanically improved connective tissue and decreased pain^23–27^. Jensen, *et al*.^28^ showed an increase in thickness of healing ligaments in rats after prolotherapy and there is evidence of rapid and sustained reductions in pain and disability for Achilles tendinopathy^29^. Next to an increase in the relative number of proliferating cells, a high glucose diet potentially also favours an increase in extracellular matrix synthesis and deposition. Singh, *et al*.^30^ report increased collagen-1 synthesis by cardiac fibroblasts *in vitro* in response to high-glucose and an enhanced deposition of ECM proteins has also been observed for cultured mesangial cells and human renal fibroblasts^31–33^.

Increased tendon thickness is also observed in tendinopathy, a chronic painful tendon disease hallmarked by loss of fibre orientation, hypervascularity and nerve ingrowth. The increase if thickness in tendinopathy may be due to increased cell proliferation and swelling. Successful therapeutic interventions like eccentric loading were shown to reduce both tendon thickness and vascular ingrowth^34, 35^.

Finally, increased tendon thickness has also been reported during chronic hyperglycaemia^36^, however the underlying mechanisms elicited by long-term elevation of blood glucose levels and concomitant systemic inflammation are most likely different.

Chondrification and calcification processes are often-described as degenerative changes arising during tendon healing and are often a hallmark of tendinopathy^37–40^. However, so far these findings have not yet been discussed in the context of nutritional aspects. The chondrogenesis associated genes *Col2a1*, *Acan*, *Comp* and *Sox9* were significantly upregulated by a high-glucose diet after 2 and 4 weeks. *Sox9* directly regulates the type II collagen gene (*Col2a1*) during chondrogenesis and may also regulate the synthesis of aggrecan^41–43^. Indeed, we also observed an increased expression of genes encoding for the proteoglycans aggrecan and COMP, which is in agreement with what has been observed in the onset of tendinopathy^9^. COMP was found to be a positive regulator in both early and late stages of chondrogenesis and has been found to be highly expressed in both developing and mature cartilage^44–46^.

Immunohistochemical stainings and gene expression analysis of collagen type I and III reflect the natural healing process, where type III collagen synthesis increases during the early phase of tendon repair and remodelling and decreases as the delayed type I collagen production outbalances and contributes to a higher organisation of the ECM^47^ (Fig. 5).

Regarding the expression of the tendon-associated genes *Scx* and *Tnmd*, we observe the highest expression 2 weeks after injury, with a reduction after 4 weeks. In a rat model of patella tendon repair, tenomodulin has previously been shown to be upregulated 1 and 2 weeks after tendon injury, with a decrease over the following remodelling period^48, 49^. Scleraxis has been shown to play a crucial role in the formation of both tendon and cartilage. Scx + /Sox9 + progenitor cells were shown to give rise to Scx−/Sox9 + chondrocytes and Scx + /Sox9− tenocytes/ligamentocytes^50, 51^. In this regard, our data propose that a high glucose diet may favour chondrogenic differentiation, as the initially high expression of scleraxis is decreased after 4 weeks, whereas the expression of *Sox9* is increased (Fig. 7). Mohawk, a transcription factor involved in early tendon development, is decreased in all repair tissues at both time points compared to intact tendon. The slight relative increase after 4 weeks in the glucose group does not necessarily reflect tenogenic differentiation, particularly in regard of *Scx* and *Tnmd* expression^52^.

Although Lin, *et al*.^8^ described elevated mRNA expression of *Sox9*, *Col2a1* and *Acan* in a rat Achilles tenotomy model without intervention, the increase in mRNAs encoding chondrogenesis-associated marker proteins we observed for the treatment group suggests that a diet rich in glucose can further enhance the formation of fibrocartilaginous nodules during tendon healing, potentially increasing the risk of tendon degeneration and/or failure. Interestingly, Topol, *et al*.^53^ also proposed a chondrogenic effect of prolotherapy in knee osteoarthritis.

As glucose metabolism affects a variety of cellular and molecular systems, it seems very unlikely that the observed effects of high-glucose on tendon repair are caused by a single molecular cascade. Generally, glucose has an essential role in the development and maintenance of musculoskeletal tissues such as bone and cartilage^54, 55^. Glucose was shown to promote chondrogenic differentiation of ATDC-5 cells by inhibiting AMP-activated protein kinase (AMPK), an essential regulator of cellular energy homeostasis. Interestingly, activation of AMPK also leads to activation of EGR-1, a transcription factor essential for tendon formation^56, 57^. In *in vitro* tissue engineering approaches for the generation of hyaline cartilage it was also shown that high glucose is essential for cartilage formation, together with insulin and dexamethasone^58^. In contrast, it was demonstrated that maintaining a low glucose environment during cell expansion is important to support chondrogenesis of human mesenchymal stem cells, by activating protein kinase C and driving the expression of TGFßRII^59^. Although this work has some limitations, our findings offer insight into how nutritional glucose can affect tendon healing after injury. Generally, a precise resection of tendon tissue of an otherwise healthy tendon is different from the ruptured Achilles tendon in patients, with its frayed ends and the possibility of preceding degenerative changes within the tendon tissue prior to rupture. However, the animal model used provides a high level of reproducibility and avoids potential confounding factors such as pre-damaged tissue or surrounding damage caused by for example ECM-degrading enzymes.

In summary, we show that a high-glucose diet following Achilles tendon injury profoundly affects tendon healing on the functional as well as on the molecular level. By favouring chondrogenic differentiation, short-term high blood glucose levels potentially pave the way for long-term tendon degeneration after injury.

## Electronic supplementary material

Supplementary Information
