# Supplementary material for: A high-glucose diet affects Achilles tendon healing in rats
Source: Sci Rep. 2017 Apr 10;7:780. doi: 10.1038/s41598-017-00700-z (PMC5429625; doi:10.1038/s41598-017-00700-z)
Supplement: Supplementary file 1 — Supplementary Information [file 41598_2017_700_MOESM1_ESM.pdf]

SUPPLEMENTARY IMFORMATION

A high-glucose diet affects Achilles tendon healing in rats

Stefanie Korntner[1,2], Nadja Kunkel[1,2,3], Christine Lehner[1,2], Renate Gehwolf[1,2], Andrea Wagner[1,2], Peter Augat[4], Daniel Stephan[4], Verena Heu[5], Hans-Christian Bauer[1,2], Andreas Traweger[1,2]†, Herbert Tempfer[1,2]†*

[1] Institute of Tendon & Bone Regeneration, Paracelsus Medical University, Spinal Cord Injury and Tissue Regeneration Centre Salzburg, AT

[2] Austrian Cluster for Tissue Regeneration, Vienna, AT

[3] University Hospital of Salzburg, Department of Trauma Surgery and Sports Injuries, Salzburg, AT

[4] Institute of Biomechanics, Trauma Center Murnau, DE

[5] University Hospital of Salzburg, Department of Paediatrics, Salzburg, AT

† contributed equally to this work

* corresponding author

Animal study design and surgical procedure

All animal experiments and procedures were conducted in accordance with Austrian laws on animal experimentation and were approved by Austrian regulatory authorities (permit numbers 20901-TVG/62/4-2013 and BMWFW-66.012/0035-WF/V/3b/2014). A total of 60 female Lewis rats aged 2 months (LEW/Crl, strain code 004; 176-200 grams) were purchased from Charles River Laboratories (Sulzfeld, Germany). They were maintained on a rotating 12/12 h light/dark cycle with an ambient temperature of 24 ± 1 °C and were provided free access to standard rat chow and water *ad libitum*, before they underwent surgery.

For creating tendon defects, rats were anesthetised via intramuscular injection of fentanyl (0.005 mg/kg bodyweight, BW; Janssen-Cilag Pharma, Vienna, Austria), midazolamhydrochloride (2 mg/kg BW; Actavis Group PTC, Hafnarfjördur, Iceland), medetomidinehydrochloride (0.15 mg/kg BW; Narco START, Richter Pharma, Wels, The Netherlands) into the left hind limb. Before surgery, each animal was given an antibiotic (enrofloxacin, 10 mg/kg) and the analgesic meloxicam (1 mg/kg). During surgery all animals continuously received oxygen and were warmed using an electric heating pad to prevent hypothermia (Harvard Apparatus; Holliston, MA). The skin was shaved and after aseptic preparation for surgery a 0.5 cm skin incision was made on the caudolateral side of the right hind limb. The Achilles tendon was carefully exposed (Fig 1a) and a 2 mm full-thickness transverse tenotomy was created using a positioning device (micro serrefine, jaw width: 2 mm) and scissors (Fig 1b). The tendon was transversally excised and 2 mm in proximal/distal extension were removed 3 mm proximal of the calcaneus resulting in a complete separation of the proximal and distal segments (Fig 1c-d). The tendon defect itself remained untreated, only the skin covering the defect was sutured using a coated vicryl (Polyglactin 910, 5-0). After the surgery, anaesthesia was antagonised by a subcutaneous injection of atipamezole (0.75 mg/kg BW; Fresenius Kabi Austria, Graz, Austria), flumazenil (0.2 mg/kg BW; Amomed Pharma, Vienna, Austria), and naloxone (0.12 mg/kg). Animals received a daily subcutaneous injection of meloxicam (1 mg/kg BW; 1x daily, Boehringer Ingelheim Vetmedica GmbH, Ingelheim/Rhein, Germany) for post-operative analgesia two days after surgery.

Immediately after surgery the feeding regimen commenced and the experimental groups received either a control diet or a high-glucose diet (ssniff® EF R/M High glucose), containing an elevated total content of carbohydrates composed of glucose instead of long chain carbohydrates, for a period of 2 (control group: n=24 / high-glucose group: n=25) and 4 weeks (control group: n=5 / high-glucose group: n=6), respectively (SI Tab 1). The control diet was composed as follows: 61 kJ% carbohydrates (46.8 starch, 10.8 sugar), 30 kJ% protein and 9kJ% fat. The high-glucose diet was composed as follows: 67 kJ% carbohydrates (50.0 glucose, 12.0 dextrines), 27 kJ% protein and 6kJ% fat. The animals were weighed prior to as well as 1, 2, 3 and 4 weeks after surgery (SI Fig 2a+b) and blood glucose was measured prior to and 2 and 4 weeks after surgery using a blood glucose meter

(SmartSystem2, Wellion, Med Trust, Marz, Austria) (SI Fig 2c). Animals were housed in groups, had access to food and water *ad libitum* and were frequently monitored for any complications or abnormal behaviour. No immobilisation was applied after surgery and rats were allowed unrestricted activities in their cages. The sutures were removed 7 days post op. After a period of 2 weeks animals were euthanised and after tendon size measurements, tendon repair tissue as well as contralateral control tendons were harvested and either stored at -20°C for biomechanical testing, immersion-fixed in phosphate-buffered saline (PBS) containing 4% paraformaldehyde for histologic analysis or stored in RNAlater (RNA stabilising reagent, Quiagen, Germany) for gene expression analysis. After a period of 4 weeks, animals were euthanised and blood samples of animals of the control group (n=5) and of the high-glucose group (n=5) were collected by cardiac puncture into tubes containing EDTA. Blood samples served for determination of glycated haemoglobin A1c (HbA1c) using a rat Hb A1c kit (Cat. No. 80300, Crystal Chem INC, Spain) according to the manufacturer's instructions (SI Fig 2d). After tendon size measurements in situ, tendon repair tissue as well as contralateral control tendons were collected for histologic analysis and gene expression analysis.

Gait analysis

Gait analysis was conducted using the CatWalk system (CatWalk XT; Noldus Information Technology, Wageningen, the Netherlands), a tool for quantitative assessment of gait and locomotion. The system consists of a walkway floor containing a sheet of glass encased with a fluorescent light. The optical properties of the glass result in internal reflectance of the light unless an external contact is made with the glass. When the rat traverses the corridor, pressure is applied to the face of the glass, the light changes the dynamic property of the glass and allows the light to escape internal reflectance, illuminating the source of pressure with luminescence proportional to the intensity of pressure [1,2]. Rats were placed in a corridor (8 cm) on a walkway (130 x 68 x 152 cm) and were allowed to move freely. A mounted camera captured a 40 cm x 10 cm field from below the walkway. As the rat entered the field of view, a run was initiated and stored on a computer. Statistics are calculated related to print dimensions and the time and distance relationships between footfalls. Rats were trained in order to get accustomed to the Catwalk. The Catwalk system was previously used to analyse the gait pattern in rats after a transverse partial transection of the Achilles tendon [3,4]. Gait analysis was conducted at three time points (pre op, 1 and 2 weeks post op). Following run criteria were defined: a run-duration of minimum 0.5 sec and maximum 5 sec, a minimum number of 5 compliant runs to acquire and a maximum allowed speed variation of 60% (run compliant). The time point before surgery included one and each of the 2 conducted time points after surgery included two trials with 5 compliant runs each. Furthermore, runs were only considered if the rat did not change direction, stop or in any other way did not satisfactorily cross the field of view. Between

each trial, the walkway barrier was removed and the glass was cleaned to remove any excrement or smell from the preceding trial using standard glass cleaner. Animals receiving a control diet for 2 weeks (n=19) and animals receiving a high-glucose diet for 2 weeks (n=20) were included for catwalk analysis. Data were processed and analysed using CatWalk XT 9.0 (Noldus Information Technology). To analyse the weight-bearing of the injured limbs, the Intermediate Toe Spread (ITS) was measured. The ITS represents the distance between second and fourth toe. Decreased ITS in the treated limb indicates a compensatory gait pattern after surgery. The stronger the limb is loaded, the larger is the distance between second and fourth toe.

Biomechanical testing

After 2 weeks, Achilles tendon repair tissues as well as contralateral control tendons of the control group (n=14) and the high-glucose group (n=14) were harvested, including the calcaneal bone, tarsals and metatarsals at the distal end and the muscle-tendon complex at the proximal end of the tendon. The metatarsals and calcaneus were positioned and embedded in Technovit (Heraeus Kulzer, Wehrheim, Germany). The tendon attachment area remained unembedded. The tendon-muscle unit was fixed in a screw grip using sandpaper (grain size 100) and superglue. Specimens were tested on a universal material testing machine (Zwick, Ulm, Germany) at 15 ° loading angle at 0.1 mm/min until failure immediately after a preload of 0.5 N had been applied. Force (N) was measured with a load cell of 200 N (accuracy class 1, Gassmann und Theiss) and recorded by corresponding software (testXpert 1, Zwick, Ulm, Germany). Maximum tensile load was defined as the maximum stress at tendon failure and is expressed in newton (N). Tendon stiffness was calculated from the linear proportion of the force/elongation curve and is expressed as N/mm.

Histology & immunohistochemistry

Achilles tendon repair tissues and contralateral tendons of all experimental groups (n=3/group) in both experiments were dissected from the proximal musculo-tendinous junction to the distal tendon-to-bone attachment zone. The tendons were immersion-fixed in phosphate-buffered saline (PBS) containing 4% paraformaldehyde (overnight, 4°C). Subsequently, they were rinsed in PBS (3× for 3 h) and cryoprotected in PBS containing 15% and 30% sucrose consecutively (3 days each, at 4°C). The tissue was embedded in frozen section compound (Surgipath®, Leica, Wetzlar, Germany), and cryosections were prepared (Leica CM1950). Serial sections of 6–30µm were collected on adhesion glass slides (Superfrost Plus; Thermo Scientific, Wien, Austria), air-dried for 1h at RT and stored at −20°C until further processing. Prior to the staining procedure, slides were allowed to equilibrate to room temperature (30–60 min) and washed in PBS.

For visualisation of the different tissue structures, 7 µm thick sections were stained using Hematoxylin&Eosin stain (data not shown), Masson-Goldner's trichrome stain, Herovici's polychrome stain and Safranin O & Fast green stain, respectively, according to the manufacturer's guidelines.

Masson-Goldner's trichrome stain was used for the detection of collagen fibres in the newly formed repair tissues. After staining the nuclei with Weigert's Fe-Haematoxylin for 2 min and a subsequent rinse in 1% acetic acid to stop the staining reaction, slides were shortly incubated in 0,5% HCl-EtOH to destain the connective tissue. After a 15 min rinse in running tap water, sections were counterstained with Goldner solution I (Ponceau acid fuchsin solution; Carl Roth, Karlsruhe, Germany) for 2 minutes. After a 30 sec rinse in 1% acetic acid a 60 min incubation with Goldner solution II (Orange G; Carl Roth, Karlsruhe, Germany) was performed. After a further rinse in 1% acetic acid sections were stained with Goldner solution III (Fast Green; Carl Roth, Karlsruhe, Germany) for 30 sec. After a final rinse in 1% acetic acid, slides were dehydrated through ethanol and Roti-Histol (Carl Roth, Karlsruhe, Germany) and mounted in Roti-Histokitt (Carl Roth, Karlsruhe, Germany) [5].

Herovici's polychrome staining was performed in order to distinguish between newly formed collagen (blue) and mature collagen fibres (red) [6,7]. The polychrome solution was prepared as a mixture of 2 parts 0.1% w/v acid fuchsin in picric acid and 1 part 0.05% w/v methyl blue solution (Sigma-Aldrich) in 1% v/v acetic acid solutions at a 2:1 ratio. The slides were incubated in this polychrome solution for 5 min at room temperature, followed by incubation for 1 min in 1% acetic acid. The slides were then dehydrated through ethanol and Roti-Histol (Carl Roth, Karlsruhe, Germany) and mounted in Roti-Histokitt (Carl Roth, Karlsruhe, Germany).

Safranin-O is a special stain for detecting acid mucopolysaccharides and was used to identify possible chondrifications in the newly grown repair tissue. After a short incubation (20 sec) in fast green solution and a quick rinse in 1% acetic acid solution for 10 sec, sections were stained in 0.1% safranin O solution for 5 minutes. The slides were then dehydrated through ethanol and Roti-Histol (Carl Roth, Karlsruhe, Germany) and mounted in Roti-Histokitt (Carl Roth, Karlsruhe, Germany) [8,9]. For documentation, a light microscope (Axioplan, Carl Zeiss, Oberkochen, Germany) and digital camera (AxioCam, Zeiss) was used. Areas of beginning chondrification were determined measuring Safranin O stained areas using ImageJ (v. 1.46) 4 weeks after surgery. For each repair tissue 3 different frontal-longitudinal sections were analysed (1 section of the middle part, 1 section ventral of the middle part and 1 section dorsal of the middle part). For each section 5 consecutive images were captured spanning the entire length of the repair tissue, omitting the transition zones of original tendon stumps to repair tissue.

Immunohistochemical detection of cells positive for KI67 and staining for Collagen type 1 (COL1) and Collagen type 3 (COL3) was performed using 30µm thick cryosections of the repair tissues after 2 and

4 weeks. After a 5-min rinse in phosphate-buffered saline (PBS), slides were incubated overnight at 4°C, with the primary antibody diluted 1:500 (monoclonal rabbit anti-KI67, RM-9106-S0, Thermo Fisher Scientific; polyclonal rabbit anti-Collagen1, ab34710, Abcam; polyclonal rabbit anti-Collagen 3, ab7778, Abcam) in PBS containing 10 % donkey serum (Sigma-Aldrich, Wien, Austria), 1% bovine serum albumin (BSA; Sigma-Aldrich), and 0.5% Triton X-100 (Merck, Darmstadt, Germany). After a rinse in PBS (four times 5 min) binding sites of primary antibodies were visualised by a corresponding Alexa568-tagged antiserum (donkey anti rabbit Alexa Fluor 568, Life Technologies, Vienna, Austria) 1:500 in PBS, containing 1% BSA and 0.5% Triton X-100 (1h at RT) followed by another rinse in PBS (four times 5 min). The slides received an additional nuclear staining using 4',6-Diamidino-2 phenylindol dihydroclorid (DAPI). For that, slides were incubated 10 min (1:4,000, stock 1 mg/ml, VWR, Vienna, Austria) followed by a rinse in PBS (three times 5 min). All slides were embedded in ProLong® Gold Antifade Mountant (Thermo Fisher Scientific). Negative controls were performed by omission of the primary antibodies during incubation and resulted in no staining [10]. Immunofluorescence images were acquired using a laser scanning microscope Zeiss LSM710 (Carl Zeiss Microscopy, Jena, Germany).

All images were processed using Adobe Photoshop CS6 (Adobe Systems, San Jose, USA). Only global corrections were performed (i.e. unsharp masking) and no specific feature within an image was manipulated. For quantification of KI67-positive cells, cryosections were immunostained for KI67, as described. For each of the tendon repair tissues, sections of different sectioning planes (upper, middle, lower section plane) were randomly chosen. For every section a total of 5 non-overlapping regions within the repair tissue area were randomly chosen. Images were acquired at a magnification of x20. Total numbers of DAPI-positive as well as KI67-positive cells were counted using ImageJ and the percentages of immune-positive cells were calculated as well as cell number/mm^2 was determined counting DAPI-positive cells.

RNA isolation

Total RNA was isolated from tendon repair tissues of the control diet group and the high-glucose diet group 2 (control group: n=5; high-glucose group: n=5) and 4 weeks after surgery (control group: n=3; high-glucose group n=3) and of intact control tendons (n=3) using TRI® Reagent (Sigma-Aldrich; Vienna, Austria) according to the manufacturer's protocol with slight modifications. Tendon tissue was homogenized using TRI Reagent® (Sigma-Aldrich, Vienna, Austria) on ice using an Ultra-Turrax (IKA, Staufen, Germany). Two additional chloroform extraction steps were performed. Total RNA was precipitated for 30min at -20°C with an equal volume of ice-cold isopropanol followed by centrifugation for 30min at 13000rpm at 4°C. RNA pellets were washed in 75% EtOH, air dried and resuspended in RNase-free water supplemented with 20units Ribolock RNase inhibitor

(ThermoFisher Scientific, Vienna, Austria and stored at -80°C. RNA yield and purity were determined using a NanoDrop 2000c spectrophotometer (ThermoFisher Scientific, Vienna, Austria) and RNA integrity was verified by the Experion Automated Electrophoresis system (Biorad, Munich, Germany). A RNA with a RNA quality indicator (RQI) >7.5 was defined as intact RNA.

Quantitative RT-PCR

1µg of total RNA was reverse-transcribed using iScript RT-PCR Supermix (BioRad, Munich, Germany) according to the manufacturer's protocol. 5ng cDNA products were subsequently analysed by quantitative PCR using TaqMan Gene Expression Master Mix (Applied Biosystems, ThermoFisher Scientific, Vienna, Austria) and TaqMan® PrimeTime Predesigned qPCR assays purchased from IDT (Integrated DNA Technologies, Coralville, IA, USA) for *Sox9* (SRY box 9), *Col2a1* (collagen, type II, alpha 1), *Acan* (Aggrecan) *Comp* (cartilage oligomeric matrix protein), *Fabp2* (fatty acid binding protein 2), *Pparγ* (peroxisome proliferator-activated receptor γ) and *Runx2* (runt-related transcription factor 2), *Col1a1* (collagen type 1 α1), *Col3a1* (collagen type 3 α1), *Scx* (scleraxis), *Mkx* (mohawk), *Tnmd* (tenomodulin), *Il1b* (interleukin 1b), *Il6* (interleukin 6), *Il10* (interleukin 10) (SI Tab 1). Default amplification conditions were as followed: 50 °C for 2 min, 95 °C for 10 min, followed by 40 cycles of 95 °C for 15 s and 60 °C for 1 min using a CFX96 Touch™ Real-Time PCR Detection System (BioRad, Munich, Germany). All samples were run in technical duplicates and a minimum of two independent experiments were performed. Cq values were analysed using qBasePlus (v. 2.4; Biogazelle NV, Zwijnaarde, Belgium) and normalised relative quantities (NRQs) were calculated by normalising the data to the expression of two previously validated endogenous control genes (*Rplp0,* Ribosomal Protein Lateral Stalk Subunit P0; *Pum1,* Pumilio RNA Binding Family Member 1) as described by Vandesompele, et al. [11].

	Crude Nutrients	Control Diet	High-Glucose Diet
Components	Dry matter	95.2	95.8
	Crude protein (N x 6.25)	20.8	19.1
	Crude fat	4.2	2.9
	Crude fibre	5.0	3.0
	Crude ash	5.6	5.6
	N free extracts	59.4	66.4
	Carbohydrates	46.8 starch<br>10.8 sugar	50.0 glucose<br>12.0 dextrines
Energy	MJ/kg (kcal/kg)	18.0 (4299.23)	17.9 (4275.34)
	**Metabolisable Energy (ME)**	61 kJ% carbohydrates<br>30 kJ% protein<br>9kJ% fat	67 kJ% carbohydrates<br>27 kJ% protein<br>6kJ% fat

SI Table 1: Diet Composition (%)

	Diet	Duration of Feeding Regimen	Specimens
2 weeks feeding regimen	Control Diet	2 weeks	n=24
	High-Glucose Diet	2 weeks	n=25
4 weeks feeding regimen	Control Diet	4 weeks	n=5
	High-Glucose Diet	4 weeks	n=6

SI Table 2: Experimental groups and feeding regimen

Feeding Regimen	Diet Group	Total Animal Number	GA	Biomechanical Testing	Thickness Measurements	IHC	Gene Expression Analysis
2 weeks	Control	25*	19	14	24	2	5
2 weeks	High-Glucose	25	20	14	25	3	5
4 weeks	Control	6*	-	-	5	2	3
4 weeks	High-Glucose	6	-	-	6	3	3

*one animal died during anaesthesia, GA: Gait Analysis, IHC: Immunohistochemistry

SI Table 3: Experimental study design

Gene name	Assay ID	Primer1 (5'-3')	Probe (5'-3')	Primer2 (5'-3')	Ref.Seq.no.
Rplp0	Rn.PT.56a.44979075	CGCAAATGCAGATGGATCG	/56-FAM/CCA CAG ACA /ZEN/AAG CCA GGA CCC T/3IABkFQ/	GTTGCCTCAGTGCCTCA	NM_022402
Pum1	Rn.PT.58.5112866	TATCTTCCACTGCCGTTCG	/56-FAM/ACCACCTCC/ZEN/TTCCCTCTCTTCACA/3IABkFQ/	CCTTAGCTTTTCCTCCTCTCC	NM_001108684
Sox9	Rn.PT.58.29440750	TCTCTTCTCGCTCTCGTTCA	/56-FAM/AGCTTGCCC/ZEN/AGAGTCTTGCTGA/3IABkFQ/	AGACCAGTACCCGCATCT	XM_003750950
Col2a1	Rn.PT.56a.35175825	CAGCTCCAGGGAATCCG	/56-FAM/AGGACCAGT/ZEN/CACTCCAGTAGGACC/3IABkFQ/	GGACAGAAAGGTGACGCT	NM_012929
Acan	Rn.PT.56a.44935331	GCAAAGAAATTGTCGGGTTGG	/56-FAM/ACTTCTCAA/ZEN/ATTGCAGGGAGTGTCCA/3IABkFQ/	AATGCTCAAGACTACCAGTGG	NM_022190
Comp	Rn.PT.56a.7115939	ACAACGCATTTCGGAGCTG	/56-FAM/TGTAGAGGA/ZEN/CTTGACAGCCTTGAGCT/3IABkFQ/	TGGAAACAGATGGAGCAGAC	NM_012834
Fabp2	Rn.PT.56a.37506480	AGTGAGTTCTGTTCCATCTGC	/56-FAM/CGACGCCGA/ZEN/GTTCAAACACAACATC/3IABkFQ/	TCACAGTCAAAGAATCAAGCAAC	NM_013068
Pparγ	Rn.PT.56a.6036576	AGCAGGTTGTCTTGGATGTC	/56-FAM/CCTGGGCGG/ZEN/TCTCCACTGAGAATA/3IABkFQ/	GAATTAGATGACAGTGACTTGGC	NM_001145367
Runx2	Rn.PT.56a.45110499	CTAAATCACTGAGGCGGTCA	/56-FAM/AACCAAGAA/ZEN/GGCACAGACAGAAGCT/3IABkFQ/	GCCACTTACCACAGAGCTAT	NM_053470
Col1a1	Rn.PT.58.8986002	GAGAACCAGCAGAGCCA	/56-FAM/TGGAGACCA/ZEN/GAGAAGCCACGATGA/3IABkFQ/	GAACAAGGTGACAGAGGCATA	NM_053304
Col3a1	Rn.PT.58.35133758	CCTGGATTACCATTGTTGCC	/56-FAM/ACTGCCCCG/ZEN/TTCACCCTTTACA/3IABkFQ/	CCCTGGTGGTTCTGGAC	NM_032085
Scx	Rn.PT.58.31750069	CTCCTCCTTCTAACTTCGAATCG	/56-FAM/ACCAGAGAA/ZEN/AGTTGAGCAAAGACCGT/3IABkFQ/	CACCCAGCCCAAACAGAT	NM_001130508
Mkx	Rn.PT.58.13871338	CGTCTAGCATTAGCGAACCAA	/56-FAM/ACACCTGCA/ZEN/CTAGCGTCATCTGC/3IABkFQ/	CTTTACAAGCACCGTGACAAC	XM_214497
Tnmd	Rn.PT.58.35753845	CACCTGTCACCAAGCCATT	/56-FAM/CCATCGACC/ZEN/GCTACATCGCCATC/3IABkFQ/	GCAGAGTTCCATCTTTAGCCT	NM_022290
Il1b	Rn.PT.58.38028824	TTGTCGTTGCTTGTCTCTCC	/56-FAM/TGGCTTATG/ZEN/TTCTGTCCATTGAGGTGG/3IABkFQ/	GTGCTGTCTGACCCATGT	NM_031512
Il6	Rn.PT.58.13840513	CCTTCTGTGACTCTAACTTCTCC	/56-FAM/TCCTTCCTA/ZEN/CCCCAACTTCCAATGC/3IABkFQ/	CAGAGCAATACTGAAACCCTAGT	NM_012589
Il10	Rn.PT.58.9773600	TGGCCTTGTAGACACCTTTG	/56-FAM/TCTTCACCT/ZEN/GCTCCACTGCCTTG/3IABkFQ/	CGACGCTGTCATCGATTTCTC	NM_012854

FAM, 6-carboxyfluorescein; *ZEN,* 5' quencher; ABkFQ, Iowa Black® Dark Quencher

SI Table 4: Primer and probe sequences of used qPCR assay

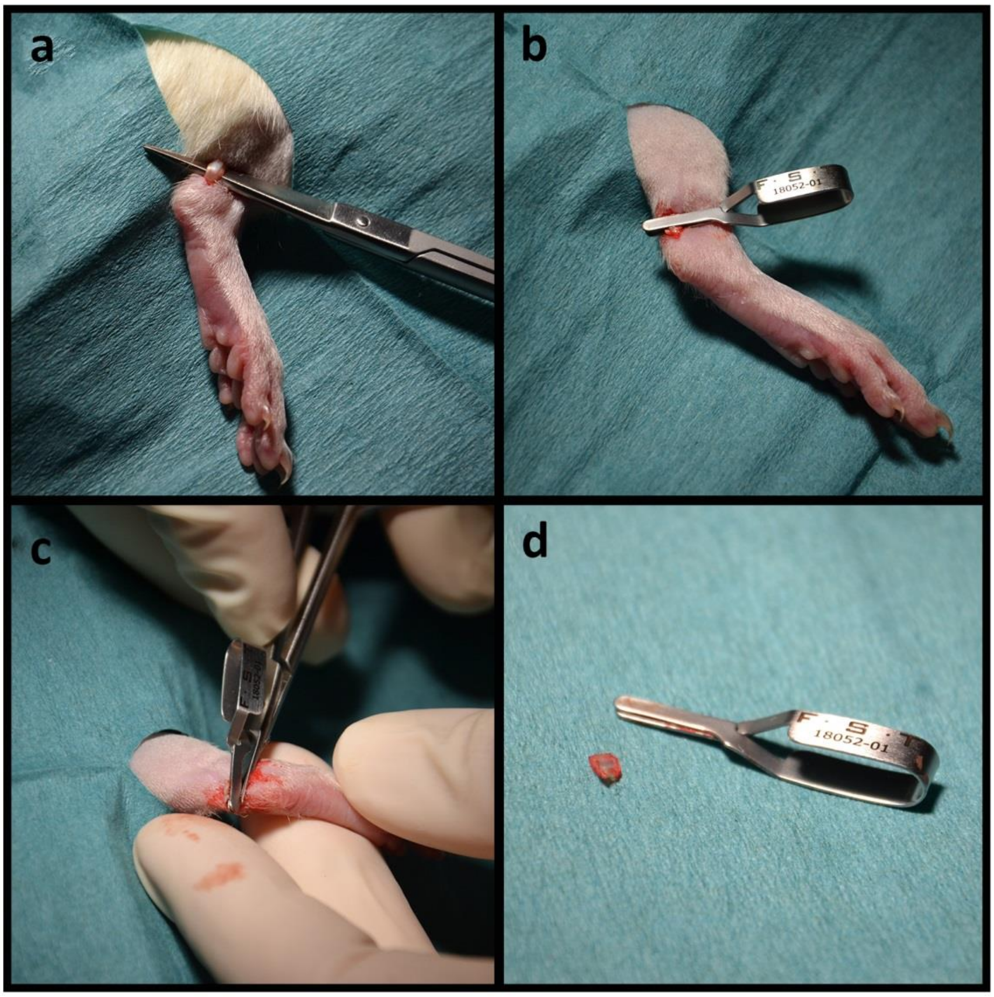

SI Figure 1: Surgical procedure of Achilles tenotomy

Intraoperative images illustrating Achilles tenotomy. The Achilles tendon was carefully exposed by a 0.5cm skin incision on the caudolateral side of the right hind limb (a) and a full-thickness defect was created using a positioning device (b) and scissors. The tendon was transversally excised (c) and 2mm in proximal/distal extension were removed (d).

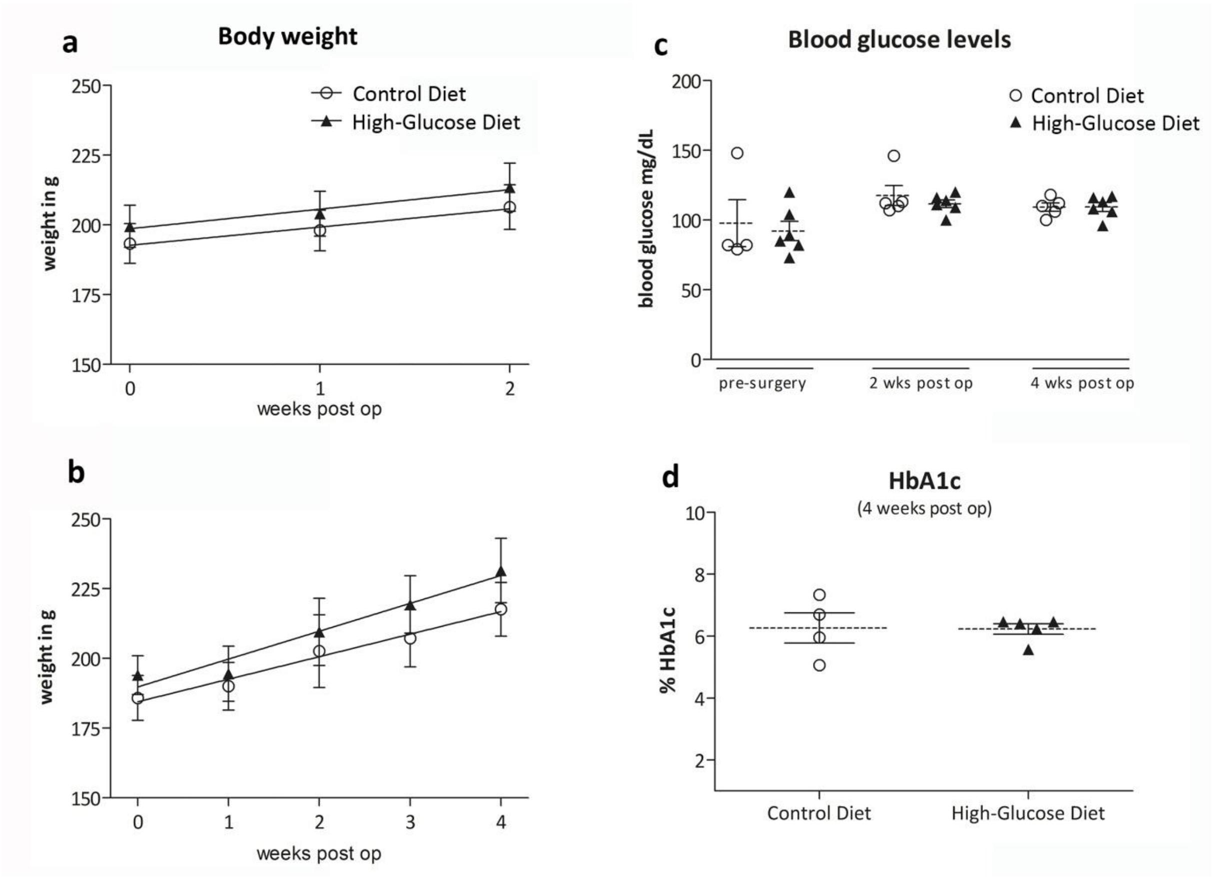

SI Figure 2: Increase in body weight over time

Body weight of animals receiving a high-glucose diet (n=25) and a control diet (n=24) over a period of 2 weeks (a). Slopes of linear regression curves are not significantly different (p=0.762; linear regression). Body weight of animals receiving a high-glucose diet (n=6) and a control diet (n=5) over a period of 4 weeks (b). Slopes of linear regression curves are not significantly different (p=0.322; linear regression). Body weight of animals was not significantly increased by a high-glucose diet neither after 2, nor after 4 weeks, compared to a control diet group. Blood glucose levels of animals receiving a high-glucose diet were in the normal range of non-diabetic individuals at all time points and comparable with the control diet group (c). HbA1c did not differ between the dietary groups after a feeding regimen of 4 weeks (p=0.90) (d).

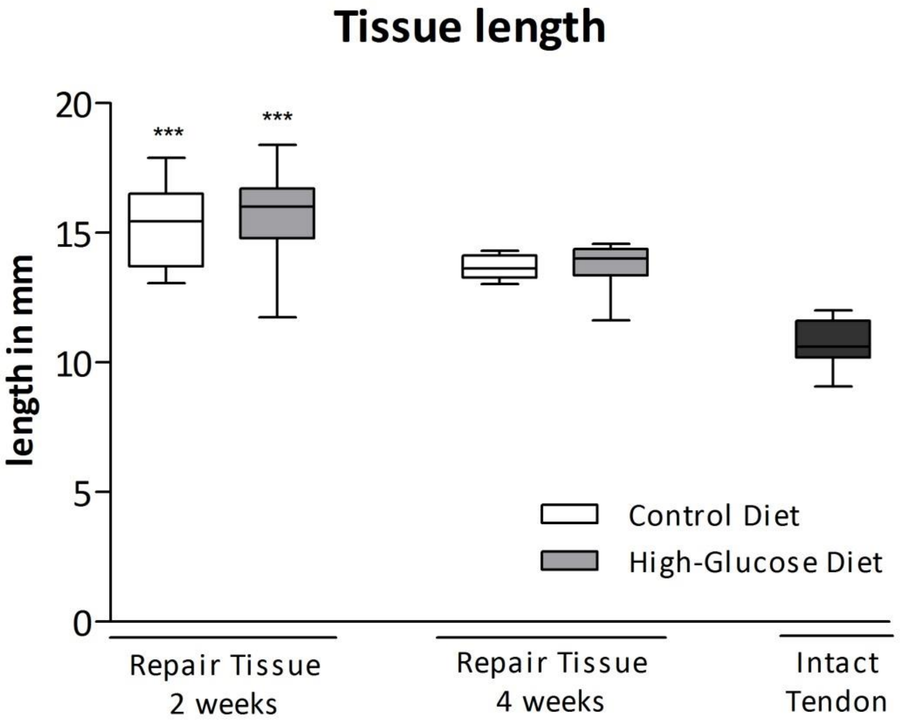

SI Figure 4: Tissue length measurements

2 weeks after surgery, repair tissue of both the high-glucose and the control group was significantly longer compared to intact tendons ($p<0.0001$; Kruskal-Wallis test & Dunn's pairwise post hoc comparisons), though there was no difference among the 2 groups (Mann-Whitney test, two-tailed). 4 weeks after surgery repair tissue length was decreased and again, no significant difference could be observed among the 2 diet groups (Mann-Whitney test, two-tailed).

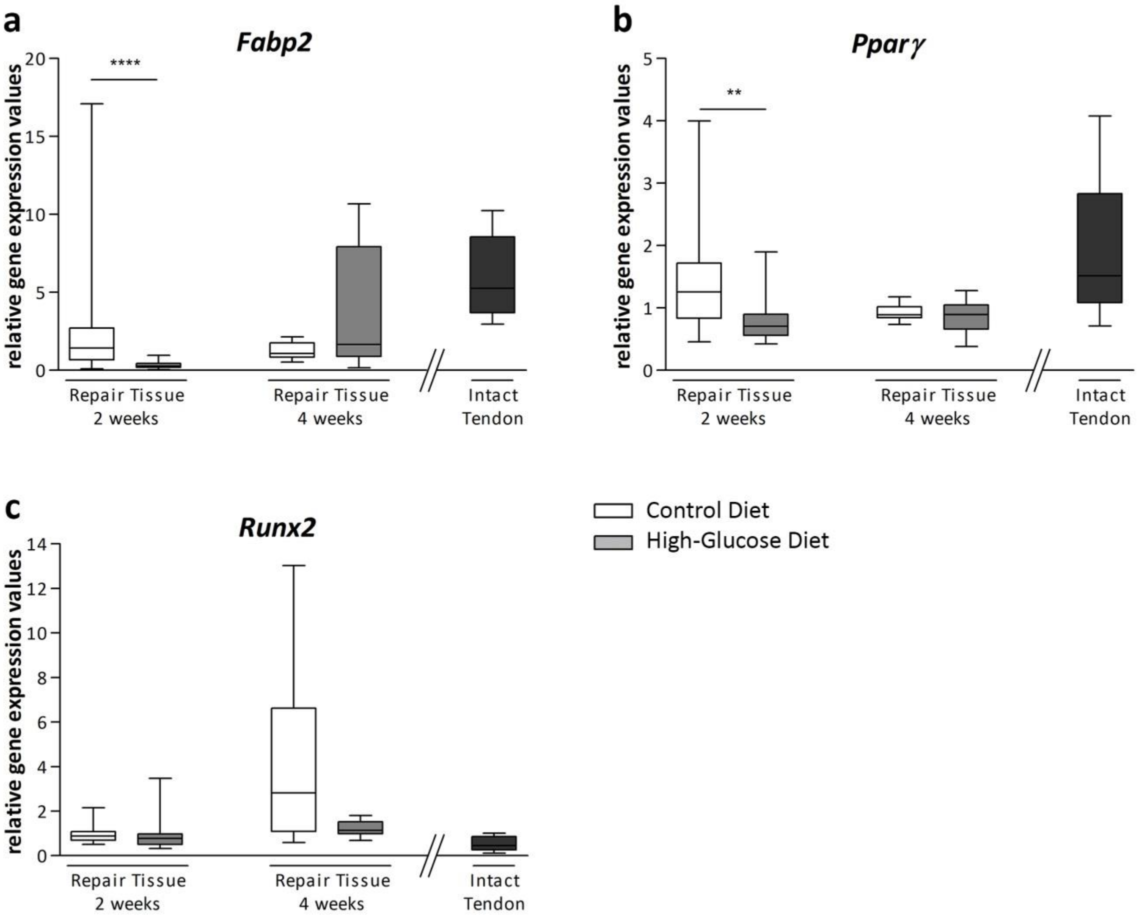

SI Figure 5: Gene expression of adipogenesis and osteogenesis-related markers

Gene expression analysis of adipogenesis- (*Fabp2, Pparγ*) (a,b) and osteogenesis-related (*Runx2*) (c) markers 2 weeks (n=5/group) and 4 weeks after surgery (n=3/group). Although a moderate repression of adipogenic and osteogenic markers was observed after 2 weeks, no significant regulation was detected after 4 weeks (Mann-Whitney test, two-tailed, Whiskers: Tukey). Values for intact tendons are provided as a reference (n=12).

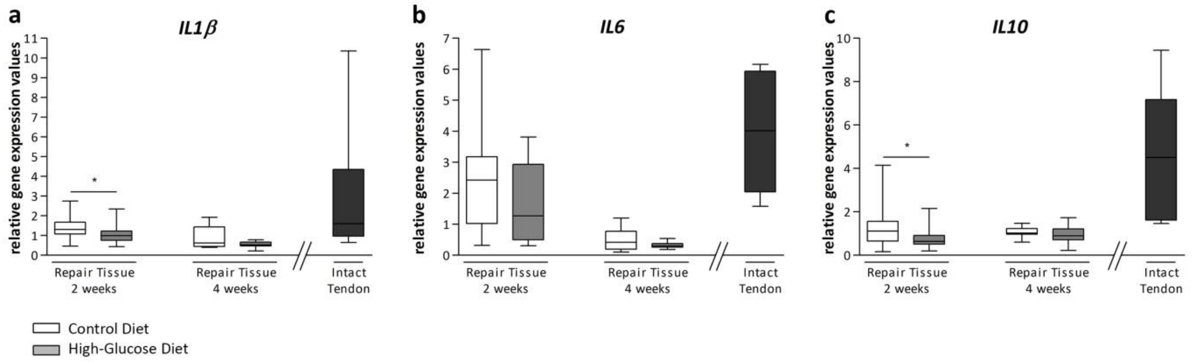

SI Figure 6: Gene expression of interleukins

Gene expression analysis of interleukin1b (a), interleukin6 (b) and interleukin10 (c) in tendon repair tissues showed no substantial regulation was detected 2 weeks (n=5/group) and 4 weeks after surgery (n=3/group) (Mann-Whitney test, two-tailed, Whiskers: Tukey). Values for intact tendons are provided as a reference.

1 Hamers, F. P., Lankhorst, A. J., van Laar, T. J., Veldhuis, W. B. & Gispen, W. H. Automated quantitative gait analysis during overground locomotion in the rat: its application to spinal cord contusion and transection injuries. *Journal of neurotrauma* **18**, 187-201 (2001).
2 Hamers, F. P., Koopmans, G. C. & Joosten, E. A. CatWalk-assisted gait analysis in the assessment of spinal cord injury. *Journal of neurotrauma* **23**, 537-548 (2006).
3 Guerra, R. F. D. *et al.* Pulsed LLLT improves tendon healing in rats: a biochemical, organizational, and functional evaluation. *Lasers in Medical Science* **29**, 805-811, doi:10.1007/s10103-013-1406-2 (2014).
4 Aro, A. A. *et al.* Arrabidaea chica extract improves gait recovery and changes collagen content during healing of the Achilles tendon. *Injury* **44**, 884-892, doi:10.1016/j.injury.2012.08.055 (2013).
5 Aescht, E. *et al. Romeis Mikroskopische Technik*. (2010).
6 Herovici, C. [Picropolychrome: histological staining technic intended for the study of normal and pathological connective tissue.]. *Revue francaise d'etudes cliniques et biologiques* **8**, 88-89 (1963).
7 Turner, N. J., Pezzone, M. A., Brown, B. N. & Badylak, S. F. Quantitative multispectral imaging of Herovici's polychrome for the assessment of collagen content and tissue remodelling. *Journal of tissue engineering and regenerative medicine* **7**, 139-148, doi:10.1002/term.508 (2013).
8 Rosenberg, L. Chemical basis for the histological use of safranin O in the study of articular cartilage. *The Journal of Bone & Joint Surgery* **53**, 69-82 (1971).
9 Tran, D. *et al.* Hematoxylin and safranin O staining of frozen sections. *Dermatologic surgery : official publication for American Society for Dermatologic Surgery [et al.]* **26**, 197-199 (2000).
10 Tempfer, H. *et al.* Presence of lymphatics in a rat tendon lesion model. *Histochemistry and cell biology*, doi:10.1007/s00418-014-1287-x (2014).
11 Vandesompele, J. *et al.* Accurate normalization of real-time quantitative RT-PCR data by geometric averaging of multiple internal control genes. *Genome biology* **3**, Research0034 (2002).
